# Supplementary material for: Predicting Remaining Survival of Glioblastoma Patients with Radiomics Analysis Based on 18F-DOPA PET Images
Source: Cancers (Basel). 2025 Nov 3;17(21):3560. doi: 10.3390/cancers17213560 (PMC12607425; doi:10.3390/cancers17213560)
Supplement: Supplementary file 1 [file cancers-17-03560-s001.zip › cancers-3881629-supplementary.pdf]

cancers

Supplementary Materials

Predicting Remaining Survival of Glioblastoma Patients with Radiomics Analysis based on ^{18}F-DOPA PET Images

Jing Qian, Deanna Hasenauer, William G. Breen, Paul D. Brown, Christopher H. Hunt, Mark S. Jacobson, Derek R. Johnson, Timothy J. Kaufmann, Bradley J. Kemp, Sani H. Kizilbash, Val J. Lowe, Michael W. Ruff, Jann N. Sarkaria, Joon H. Uhm, Mark J. Zakhary, Maasa H. Seaberg, Hok Seum Wan Chan Tseung, Elizabeth S. Yan, Yan Zhang, Nadia N. Laack and Debra H. Brinkmann

Table S1. The extracted features in this study include 26 shape, 19 first-order and 70 texture features from each scan. And the texture features can be furthered categorized into 24 gray level co-occurrence matrix (GLCM), 16 gray level dependence matrix (GLDM), 16 gray level run length matrix (GLRLM), and 14 gray level size zone matrix (GLSZM)).

Feature Categories	Feature Subcategories	Feature Names
Shape		Elongation, Flatness, LeastAxisLength, MajorAxisLength, Maximum2DDiameterColumn, Maximum2DDiameterRow, Maximum2DDiameterSlice, Maximum3DDiameter, MeshVolume, MinorAxisLength, Sphericity, SurfaceArea, SurfaceVolumeRatio, VoxelVolume
First order		10Percentile, 90Percentile, Energy, Entropy, InterquartileRange, Kurtosis, Maximum, MeanAbsoluteDeviation, Mean, Median, Minimum, Range, RobustMeanAbsoluteDeviation, RootMeanSquared, Skewness, TotalEnergy, Uniformity, Variance
Texture	gray level co-occurrence matrix (GLCM)	Autocorrelation, ClusterProminence, ClusterShade, ClusterTendency, Contrast, Correlation, DifferenceAverage, DifferenceEntropy, DifferenceVariance, Id, Idm, Idmn, Idn, Imc1, Imc2, InverseVariance, JointAverage, JointEnergy, JointEntropy, MCC, MaximumProbability, SumAverage, SumEntropy, SumSquares
	gray level dependence matrix (GLDM)	DependenceEntropy, DependenceNonUniformity, DependenceNonUniformityNormalized, DependenceVariance, GrayLevelNonUniformity, GrayLevelVariance, HighGrayLevelEmphasis, LargeDependenceEmphasis, LargeDependenceHighGrayLevelEmphasis,

	LargeDependenceLowGrayLevelEmphasis, LowGrayLevelEmphasis, SmallDependenceEmphasis, SmallDependenceHighGrayLevelEmphasis SmallDependenceLowGrayLevelEmphasis,
gray level run length matrix (GLRLM)	GrayLevelNonUniformity, GrayLevelNonUniformityNormalized, GrayLevelVariance, HighGrayLevelRunEmphasis, LongRunEmphasis, LongRunHighGrayLevelEmphasis, LongRunLowGrayLevelEmphasis, LowGrayLevelRunEmphasis, RunEntropy, RunLengthNonUniformity, RunLengthNonUniformityNormalized, RunPercentage, RunVariance, ShortRunEmphasis, ShortRunHighGrayLevelEmphasis, ShortRunLowGrayLevelEmphasis
gray level size zone matrix (GLSZM))	GrayLevelNonUniformity, GrayLevelNonUniformityNormalized, GrayLevelVariance, HighGrayLevelZoneEmphasis, LargeAreaEmphasis, LargeAreaHighGrayLevelEmphasis, LargeAreaLowGrayLevelEmphasis, LowGrayLevelZoneEmphasis, SizeZoneNonUniformity, SizeZoneNonUniformityNormalized, SmallAreaEmphasis, SmallAreaHighGrayLevelEmphasis, SmallAreaLowGrayLevelEmphasis, ZoneEntropy, ZonePercentage, ZoneVariance

The detailed definition of the features can be found in https://pyradiomics.readthedocs.io/en/latest/features.html (accessed on 5 August 2025).

Table S2. The list of identified delta features showing significant differences between preRT and postRT images, with a brief description of each feature.

Category	Features	Simple Description
Shape	MeshVolume	Total tumor volume calculated with a triangle mesh.
	MajorAxisLength	The longest axis of the ellipsoid enclosing the Region Of Interest (ROI) and is computed as $4\sqrt{\lambda_{major}}$ where λ_{major} is the largest eigenvalue from Principal Component Analysis (PCA). The PCA is performed on the physical coordinates of ROI pixel/voxel centers (thus spacing-aware) and does not rely on a surface mesh.
	LeastAxisLength	The shortest axis of the ellipsoid enclosing the ROI, computed as $4\sqrt{\lambda_{least}}$, where λ_{least} is the smallest eigenvalue from PCA performed on the physical

		coordinates of ROI voxel centers (spacing-aware, mesh-independent).
First Order	Variance	The dispersion of voxel intensities within the ROI as the mean squared deviation from the mean. By definition, this equals the squared standard deviation.
Texture	[GLCM] Correlation	GLCM Correlation captures how predictably neighboring voxels' intensities vary together: values near 1 mean the gray levels rise and fall in a consistent, linear way, while values near 0 indicate little to no relationship (more random texture). In short, higher correlation = more coordinated intensity patterns across neighbors.
	[GLRLM] LongRunEmphasis	GLRLM Long Run Emphasis (LRE) reflects how much the image contains long stretches of the same gray level. A higher LRE means larger, more uniform patches, i.e., a coarser-looking texture with fewer short, fine details.

The detailed description and mathematical definition of the features can be found in https://pyradiomics.readthedocs.io/en/latest/features.html (accessed on 5 August 2025).

Table S3. Feature count reduction flow in the feature processing steps utilized in this study.

Feature Processing	Feature Selection Step	Feature count
Extraction	N/A	115
Selection	Individual Predictivity Evaluation	104
	Predictivity cross validation	47
	Category-based Pair Correlation	6
Manifold learning	N/A	2
